# Interradicular trabecular bone density of the lateral maxilla for temporary anchorage devices – a histomorphometric study

**DOI:** 10.1186/s13005-015-0058-9

**Published:** 2015-02-07

**Authors:** Elena Krieger, Heinrich Wehrbein

**Affiliations:** Department of Orthodontics, Medical Centre of the Johannes-Gutenberg-University Mainz, Augustusplatz 2, 55131 Mainz, Germany; Department of Orthodontics, Medical Centre of the Johannes-Gutenberg-University Mainz, Augustusplatz 2, 55131 Mainz, Germany

**Keywords:** Orthodontic, Anchorage, Implant, Insertion site, Failure rate, Bone density

## Abstract

**Objective:**

To analyze the interradicular trabecular bone density of the lateral maxilla regarding the insertion of temporary anchorage devices (TADs).

**Material and methods:**

The material consisted of tissue blocks of autopsy material from 20 subjects (17 male, 3 female, 16 - 63y). The specimens comprised the dentated alveolar bone of the lateral maxilla. The interradicular areas (IRA) from canine to distally of the second molar (IRA 3–4, 4–5, 5–6, 6–7, 7d) were histomorphometrically measured with respect to the hard tissue fraction of the trabecular bone (HTFTB, %) and statistically analyzed.

**Results:**

Histomorphometric measurements showed the following results: Mean HTFTB of IRA 3–4 was 44.08%, of IRA 4–5 31.07%, of IRA 5–6 33.96%, of IRA 6–7 36.33% and of IRA 7d 25.40%. Only the difference between the HTFTB of IRA 3–4 and the other IRAs was statistically significant (*p < 0.05*). Regarding the minimum and maximum HTFTB value of each IRA, there was a great amount of difference, especially for IRA 3–4: minimum HTFTB was 17.20% and maximum 67.03%.

**Conclusion:**

Apart from the IRA between canine and first premolar, the HTFTB in the IRAs of the lateral maxilla have to be classified as low or even moderate. IRA 3–4 should also be considered cautious regarding its minimum values. Thus, it seems that the interradicular trabecular bone density of the lateral maxilla is unfavorable to achieve a good primary stability of TADs.

## Introduction

Temporary anchorage devices (TADs) are nowadays commonly used as anchorage for orthodontic tooth movement. They are temporarily inserted, and after accomplishing the treatment purposes removed. Several devices were developed and three types of TADs are usually applied: mini-plates (i.e. bone anchor), length-reduced mini-implants (i.e. palatal implant) and diameter-reduced mini-implants (i.e. mini-screw). Each type has its specific insertion area with individual failure rates, also including parameters of the individual patient [1].

The initial bone-implant-interface is highly important and influenced by the bone quality and quantity, the implant geometry, and the site preparation technique [2]. Therefore, the bone quantity and quality of the specific insertion area is of major interest. Recently, several studies have been published investigating the bone density concerning orthodontic treatment and TADs by using computed tomography (CT) or cone beam computed tomography (CBCT) [3-7]: Samrit et al. [3] evaluated the bone density in interradicular bone between second premolars and first molars and its association with the clinical stability of mini-screws used for en masse retraction of anterior teeth in 10 extraction cases. A comparison between maxilla and mandible revealed higher values in mandibular cortical bone and no difference in cancellous bone values [3]. Kim and Park [4] measured the cortical bone thickness in the mandibular buccal and lingual areas in order to assess the suitability of these areas for application of TADs. Chugh et al. [5], Chun and Lim [6], as well as Cassetta et al. [7] evaluated the alveolar cortical bone thickness and density differences between interradicular sites at different levels from the alveolar crest. All authors found differences in bone densities depending on the localization, anterior to posterior areas and from crest to base of the alveolar crest.

Marquezan et al. [8] compared with micro-CTs the primary stability of mini-screws inserted into bovine bone blocks of different densities with and without cortical bone, and investigated if trabecular properties could influence primary stability. They found that trabecular bone had an important role in primary stability in the presence or absence of cortical bone.

But evaluating the bone quantity histomorphometrically, only a few studies can be found: Wehrbein [9] assessed quantitatively the bone quality of the palatal bone of 22 human tissue blocks of autopsy material. He suggested a good primary stability of TADs inserted in this area [9]. Çehreli and Arman-Özçırpıcı [10] evaluated the primary stability and histomorphometric measurements of 72 mini-screws inserted in bovine iliac crest blocks. They found positive correlations between the bone-implant contact and cortical bone densities [10].

Thus, this is the first study assessing the interradicular trabecular bone density of the lateral maxilla regarding the insertion of temporary anchorage devices (TAD) histomorphometrically in humans.

## Material and methods

The material consisted of tissue blocks of autopsy material from 20 subjects (17 male, 3 female), between 16 and 63 years of age. The specimens comprised tooth-bearing lateral segments of the maxilla from the canine to the second molar region. Inclusion criterion was that all observed teeth had to have an antagonist in the mandible; therefore, all teeth were functionally loaded during lifetime (i.e. mastication). Further inclusion criteria were the absence of crowns or bridges. The specimens were obtained from the Institute of pathology and the Institute of forensic medicine at the University of Aachen after the required authorization was given by the legally responsible person. According to the information given to us no diseases other medical conditions concerning the bone metabolism were present.

**Figure 1:**
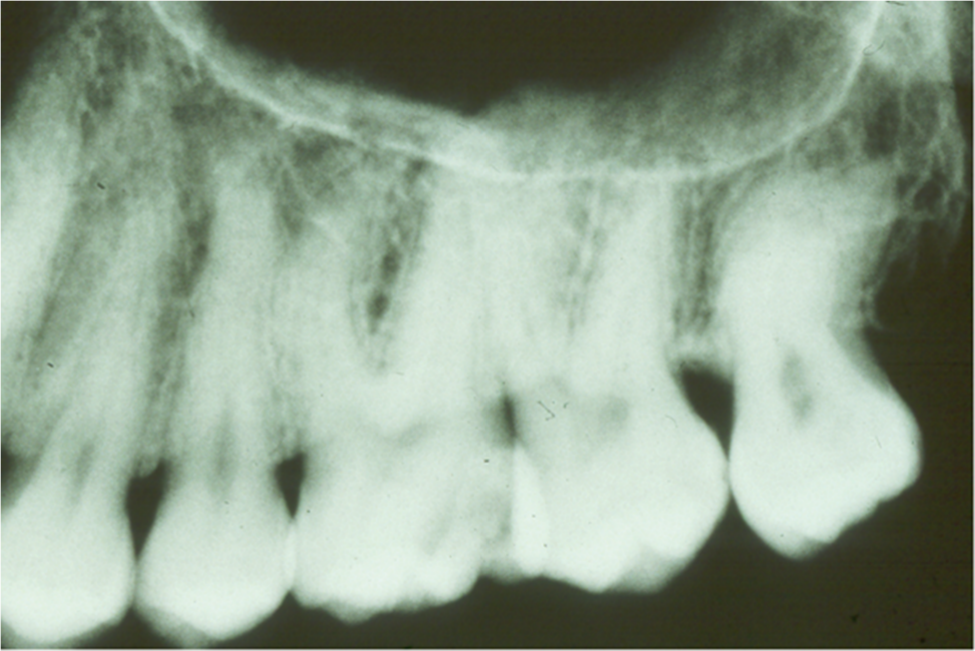
**Radiographic of a specimen showing the interradicular areas.**

The following interradicular areas (IRA) were measured (Figure 1): between canine and first premolar (IRB 3–4); first and second premolar (IRB 4–5); second premolar and first molar (IRB 5–6); first and second molar (IRB 6–7); distal end of the second molar (IRB 7d).

### Histomorphometry

All specimens were assessed histomorphometrically. This procedure is by definition a quantitative study of the microscopic organization and structure of tissue (for example bone) particularly by means of computer-assisted analysis of images formed by a microscope.

**Figure 2:**
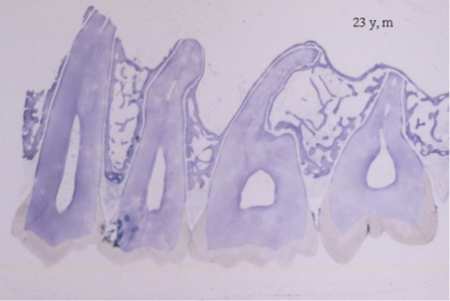
**Histological sample of a maxilla segment of a 23 y old male specimen: overview of the IRAs; the trabecular structures are evenly distributed (original magnification).**

**Figure 3:**
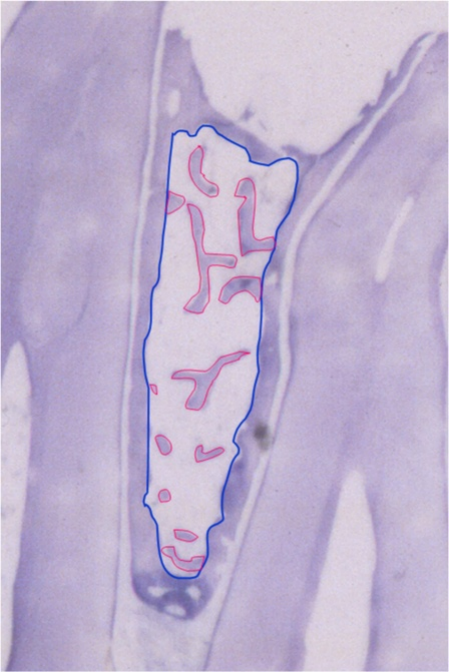
**Magnification of the interradicular bone between first and second premolar of Figure** 2 **: relatively low trabecular bone density (magnification × 2.5).**

The histological sections were prepared in the sagittal plane according to the ground-thin-section technology according to Donath 1988 [11] (Figure 2). Accordingly three series of slices from the middle section of the maxillary segments (5–20 μm thickness) were stained with toluidine blue (Figure 3). The evaluation of the specimen was performed by using the semiautomatic method for quantitative static and dynamic bone histology by Malluche et al. [12]: a microscope is equipped with a drawing tube through which the image of the digitizing platen is projected over the optical field; the investigator selects and traces all histologic structures to be measured by moving a cursor on the digitizing platen which is visible by its projection over the histologic field. Reliability and accuracy were shown by Malluche et al. [12].

The histomorphometry was carried out in our study with a computer (IBM, Armonk, USA), a SummaSketch digitizer and a digitizing tablet (Summagraphics Corporation, Lansdale, Pennsylvania USA) along with the appropriate software and a microscope (Leica Microsystems, Wetzlar, Germany). The magnification used was 390.6× . The microscope bearded a grid, which was subdivided into 25 squares. Each square had a side length of 435.89 μm. The grid was placed in each IRA and centered at the level of the apices. Of each specimen, ten of the 25 squares were analyzed and the trabecular bone areas measured. The measured trabecular bone areas in relation to the total surface of the ten squares (i.e. total bone area = 1.900.000 μm^2^) lead to the hard tissue fraction of the trabecular bone in percentage (HTFTB, %).

### Statistics

Statistical analysis was performed by using the SAS program. Mean, minimum, maximum values and standard deviations were calculated for each IRA after computing the average value from the measurement of the three slices. Data of each IRA were compared using the Wilcoxon-test. A p-value was calculated and considered as statistically significant when *p < 0.05*.

## Results

### Location-specific bone quality

The histomorphometric measurements of the HTFTB of all interradicular areas are shown in Table 1. For example, The IRA 3–4 had a HBTFB of 44.08%, meaning a density of trabecular bone of 44.08% in relation to the total bone area (1.900.000 μm). The lowest mean HTFTB was found in the IRA 7d (25.40%), and the highest mean HTFTB in IRA 3–4 (44.08%).

**Table 1:** **Percentage of hard tissue fraction to total bone volume (HTFTB, %) of all interradicular areas: IRA 3–4, IRA 4–5, IRA 5–6, IRA 6–7 and IRA 7d; mean, standard deviation (SD), minimum (min), maximum (max) and variance**

	**IRA 3-4**	**IRA 4-5**	**IRA 5-6**	**IRA 6-7**	**IRA 7d**
Mean	44.08%	31.07%	33.96%	36.33%	25.40%
SD	14.32%	12.18%	12.74%	8.12%	12.13%
Min	17.20%	16.82%	20.50%	27.20%	11.20%
Max	67.03%	52.20%	62.40%	53.90%	38.60%
Variance	202.198	164.229	162.554	67.998	147.244

Regarding the minimum and maximum value of each IRA, there was a great amount of difference, especially for the IRA 3–4: its minimum value was 17.20% and maximum 67.03%.

Regarding differences between the individual IRAs, only the difference between the HTFTB of the IRA 3–4 and the others was statistically significant (*p < 0.05*) (Wilcoxon-Test).

## Discussion

Until now, investigations of the bone density of the alveolar bone taking into account to insert TADs for orthodontic treatment purposes, can only be found using CT or CBCTs for assessment [3-8,12-14]. The accordance between radiographic assessments and histomorphometric measurements was evaluated by González-García and Monje [15], but in terms of dental implants. They conducted a study to assess the reliability of CBCTs as a tool to pre-operatively determine radiographic bone density. Therefore, this is the first study assessing the interradicular trabecular bone density of the lateral maxilla regarding the insertion of TADs histomorphometrically in humans.

The primary stability of mini-screws is significantly influenced by the bone density of the cortical bone [13]. Marquezan et al. [8] reported in their micro-CT investigation of TADs in bovine bone that the presence of cortical bone increased the primary stability, and that the cortical bone had an important role when the trabecular bone had a lower bone density. We found mean HTFTB values of the lateral maxilla considering all IRAs from 25.40% (IRA 7d) up to 44.08% (IRA 3–4). The mean HTFTB values were similar from mesial to distal (between 25 and 44%). Nevertheless, the minimum values instead were between 11 and 27%. There was also a wide difference between the minimum and maximum values of each IRA, especially for IRA 3–4. The high standard deviations as well as differences between the minimal and maximum values in the respective IRAs are probably due to different loading conditions (occlusal wear) in the respective individuals than to other medical conditions as to our information no diseases or other medical conditions concerning bone metabolism were known. Also, age and gender of the specimen might have been an influence factor. Accordingly, Wakimoto et al. [16], who investigated the bone quality and quantity of the anterior maxillary trabecular bone in dental implant sites, found that women had lower bone densities than men.

Therefore, we concluded that apart from IRA 3–4 the HTFTB in the IRAs of the lateral maxilla have to be classified as low (IRA 7d) or even moderate, but IRA 3–4 should also be considered cautious regarding its minimum values. Thus, the interradicular trabecular bone density of the lateral maxilla seems to be unfavorable to achieve a good primary stability of TADs. Therefore, the cortical bone thickness and density are still decisive factors for primary stability of TADs in the lateral maxilla.

Our findings are similar to Chugh et al. [5], who investigated the cortical bone density. They reported that the highest cortical bone density was observed between the second premolar and first molar at the alveolar bone level and between the first and second molars at the basal bone level in the maxilla [5]. The maxillary tuberosity presented the least bone density which is comparable to the IRA distally of the second molar (IRA 7d) in our investigation. Chun and Lim [6] evaluated bone density differences between interradicular sites. They suggested that mini-implants for orthodontic anchorage would more be effective when placed in areas with equivalent bone density up to 6 mm apical to the alveolar crest [6].

Comparing our results to the anterior palate, where a hard tissue fraction to total bone volume of 68% was found histomorphometrically [9], it is pointed out that this region is more appropriate for the insertion of TADs. The initial and possibly the subsequent hart tissue contact of TADs inserted in the anterior palate are twice as high as in the interradicular area of the maxilla [9]. The multicenter investigation by Jung et al. [17] reported about a failure rate of 4.6% (n = 239) when inserting a length-reduces implant in the anterior palate. Regarding all TADs, the meta-analysis of Schätzle et al. [1] demonstrated the following data: the failure rate for length-reduces palatal implants was 10.5%, for mini-screws inserted in the alveolar bone 16.4% and for mini-plates 7.3%. They reported that mini-plates and palatal implants together, representing torque-resisting TADs, showed a 1.92-fold lower clinical failure rate than mini-screws [1].

TADs are inserted to support orthodontic treatment purposes. Orthodontically induced tooth movement implies changes in the surrounding tissue (hard and soft). Hsu et al. [18] assessed 2011 the bone density changes around the anterior teeth during orthodontic treatment by using CBCTs. The bone density around the teeth reduced significantly after the application of orthodontic forces [18]. This findings suggest, when inserting a TAD into alveolar bone, where recently orthodontic tooth movement was conducted, the primary stability could be even more reduced and therefore higher failure or migration rates might occur.

## Conclusions

The following conclusions can be drawn:

- Apart from the interradicular area between canine and first premolar (IRA 3–4) the mean hard tissue fraction of the trabecular bone (HTFTB) in the interradicular areas of the lateral maxilla have to be classified as low (IRA 7d) or even moderate.
- There was a wide difference between the minimum and maximum values of each IRA, especially for IRA 3–4. Therefore, IRA 3–4 should also be considered cautious regarding its minimum values.
- Thus, it seems that the interradicular trabecular bone density of the lateral maxilla is unfavorable to achieve a good primary stability of TADs. Therefore, the cortical bone thickness and density are decisive factors for primary stability of TADs in the lateral maxilla.

## Abbreviations

CBCT: Cone beam computed tomography
CT: Computed tomography
HTFTB: Hard tissue fraction of the trabecular bone
IRA: Interradicular area
TAD: Temporary anchorage device
